# Relationship between biodosimetric parameters and treatment volumes in three types of prostate radiotherapy

**DOI:** 10.1038/s41598-021-03417-2

**Published:** 2021-12-23

**Authors:** Zsuzsa S. Kocsis, Tibor Major, Csilla Pesznyák, Dalma Mihály, Gábor Stelczer, Márta Kun-Gazda, Gyöngyi Farkas, Gábor Székely, Péter Ágoston, Kliton Jorgo, László Gesztesi, Csaba Polgár, Zsolt Jurányi

**Affiliations:** 1 Department of Radiobiology and Diagnostic Onco-Cytogenetics, Centre of Radiotherapy, National Institute of Oncology, Budapest, Hungary; 2 Department of Oncology, Semmelweis University, Budapest, Hungary; 3 Centre of Radiotherapy, National Institute of Oncology, Budapest, Hungary

**Keywords:** Cancer, Immunology, Oncology, Urology, Physics

## Abstract

Brachytherapy (BT) and external beam radiotherapy (EBRT) apply different dose rates, overall treatment times, energies and fractionation. However, the overall impact of these variables on the biological dose of blood is neglected. As the size of the irradiated volume influences the biological effect as well, we studied chromosome aberrations (CAs) as biodosimetric parameters, and explored the relationship of isodose surface volumes (ISVs: V_1%_, V_1Gy_, V_10%_, V_10Gy_, V_100%_, V_150%_) and CAs of both irradiation modalities. We performed extended dicentrics assay of lymphocytes from 102 prostate radiotherapy patients three-monthly for a year. Aberration frequency was the highest after EBRT treatment. It increased after the therapy and did not decrease significantly during the first follow-up year. We showed that various types of CAs 9 months after LDR BT, 3 months after HDR BT and in a long time-range (even up to 1 year) after EBRT positively correlated with ISVs. Regression analysis confirmed these relationships in the case of HDR BT and EBRT. The observed differences in the time points and aberration types are discussed. The ISVs irradiated by EBRT showed stronger correlation and regression relationships with CAs than the ISVs of brachytherapy.

## Introduction

In addition to external beam radiotherapy (EBRT), brachytherapy (BT)—a method of radiotherapy when radioactive isotopes are placed into or close to the tumor, to exploit the steep decrease of the dose with the distance—has an expanding role in prostate cancer therapy^1^. However, biologically the BT and EBRT treatments differ substantially^2^. They use different overall treatment times and dose rates. Low dose rate (LDR) BT is carried out on a single occasion and the dose delivery is continuous. Although high dose rate (HDR) brachytherapy is most frequently used in a fractionated manner, in our centre, prostate HDR BT is given in a single fraction. In BT, different isotopes are used with various energies and dose rates. There are already numerous studies on how these different variables influence the biological effects: linear quadratic model for dose dependence and biological effective dose calculations for fractionation. However, the weight of these factors in the summarised effect is unknown^3^. The clinical results (side effects, survival) are thoroughly investigated, but the effect of irradiation on cell level for deeper understanding is often neglected. Biological dosimetry is a tool to supplement physical dosimetry for this purpose^4^.

The biological dosimetry model systems, such as blood or cell culture irradiation^5^, however, are not able to model some therapeutic modalities. The delivery of the prescribed dose in LDR therapy, for example, lasts approximately 1 year. Therefore, studies performed on humans are needed, which are available only in clinical settings. However, these studies cannot be interpreted without considering the affected volumes of irradiation, which are also different in these techniques.

Our aim was to compare biodosimetric values (chromosome aberration frequencies) with volumes enclosed by isodose surfaces in cases of EBRT and two variants of BT. We wanted to explore how their relationship differs between the modalities. Thus, we investigated the total effect of the other characteristics of the modalities (photon energy, dose rate, overall treatment time and fractionation). We also examined which time points and chromosomal abnormalities were most affected by the volumetric effect after the end of radiotherapy.

On the other hand, numerous studies showed that radiotherapy can modify the number, distribution and activity of the different immune cell populations^6^. The gaining importance of immunotherapies used in combination with radiation motivates the research on the number of immune cells destroyed or with modified activity. For example, radiation induced lymphopenia before immunotherapy decreased survival in a mixed cohort of non-small cell lung (NSCLC), renal cancer and melanoma^7^ and in another NSCLC cohort^8^. Furthermore, the nadir of absolute lymphocyte count after chemoradiotherapy was associated with both overall survival and disease specific survival loss in 504 esophageal cancer patients^9^. In addition, the low total lymphocyte count 2 months after chemoradiation of pancreatic cancer patients was an independent predictor of inferior progression free survival in multivariate analysis^10^. In RTOG 0617 the higher dose arm (74 Gy instead of 60 Gy) also showed the less local progression free survival in chemoradiotherapy treated NSCLC patients^11,12^. A computational model showed that the dose on immune cells was associated with the loss of the survival^13^. We also suggest that it is important to know the dose distribution on lymphocytes, because the white blood cells have different radiosensitivity. Although there are models^14–17^ from calculations, it is highly difficult to obtain the radiation dose of blood. Our study provides comparison between the modalities and the characteristics of the techniques could be incorporated into these models. We also obtained data on the distribution of chromosome aberrations in the lymphocyte population, therefore, our data might be suitable for testing these models.

**Figure 1:**
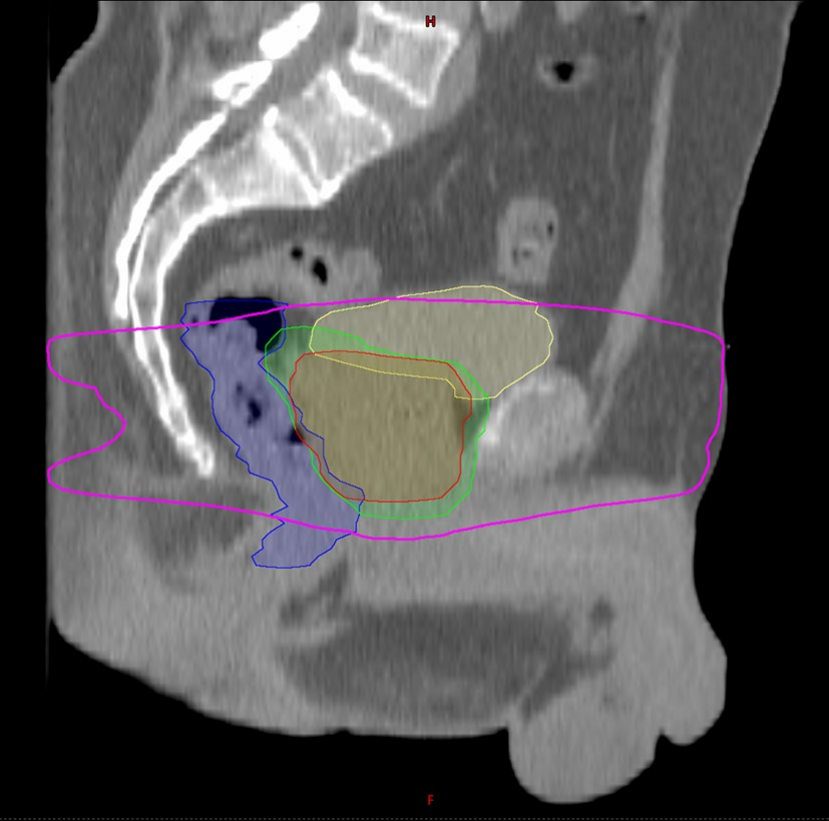
Example of an isodose surface volume (ISV) and organ related volumes of a patient with prostate cancer. The purple line indicates the border of the V_10Gy_ ISV, the red delineation represents the prostate PTV and the green delineation shows the PTV_PVS (prostate and vesicular seminalis). The rectum and bladder was indicated with blue and yellow color, respectively.

The dicentric assay is one of the most reliable conventional biodosimetric methods^4,18^. Chromosome aberrations are studied in peripheral blood lymphocytes, therefore, the organ/tissue related differences in the biology of the different treatment types can be excluded. Lymphocytes also reach every part of the body in the blood circulation and can be easily collected. However, the irradiated lymphocytes are present not only in the prostate, but in the irradiated surrounding tissues as well. Therefore, instead of the organ related volumes, we examined volumes enclosed by an isodose surface (isodose surface volume, ISV), which are organ independent, but are calculated inside the body contour only. These volumes correspond to all the irradiated volumes getting a minimum of these doses (given in cubic centimeters), regardless of the affected organs (Fig. 1). However, it should be mentioned that treatment planning systems may not calculate the isodose volumes irradiated with small doses accurately, and the phenomenon that small dose isodose curves fall outside the visual field of CT scans and ultrasound pictures must also be taken into account.

Lymphocytes, which are considered as a surrogate normal tissue in biodosimetry, flow through the irradiated volumes and are continuously replaced by newer ones. As a result, merely considering the irradiated volume may lead to erroneous dose estimation because a more significant irradiated volume does not necessarily mean a greater biological effect. In view of the above, there is a need for a study that examines the effect of irradiated volumes on biological dose in radiotherapy.

There are a lot of publications about dosimetric calculation or in vivo dosimetry in radiotherapy, but according to our knowledge publications dealing with the influence of isodose surface volumes on biological dosimetric values are still missing. Moreover, most of the analyses of volumetric data use organ related volumes, therefore they are not applicable for chromosome aberrations.

## Results

The following isodose surface volumes (ISVs) of absolute and relative doses were recorded: V_1%_, V_1Gy_, V_10%_, V_10Gy_, V_100%_, V_150%_ (Table 1) (except V_1%_ in the case of HDR therapy, discussed below). These volumes cover all the irradiated volumes getting at least a minimum of these doses, regardless of the affected organs. The variability in ISVs was high among patients. For the sake of example, the highest ratio of maximum to minimum was 3.9 for V_150%_ in HDR therapy. The higher ratios can be seen at higher doses in all therapies, but the highest differences were found mostly in HDR therapy (Table 1).

**Table 1:** Volumes enclosed by an isodose surface (ISV) in cm^3^ for three kinds of prostate radiotherapy.

Volume	LDR BT			HDR BT			EBRT		
	Mean (cm^3^)	Range (cm^3^)	Max/min	Mean (cm^3^)	Range (cm^3^)	Max/min	Mean (cm^3^)	Range (cm^3^)	Max/min
V_1%_	2296.3	1897–2878	1.5	–	–		10,580.0	8083–17,162	2.1
V_1Gy_	2533.2	2104–3108	1.5	2088.3	1336–2755	2.1	9099.0	7172–14,549	2.0
V_10%_	484.5	345–707	2.0	982.6	532–1596	3.0	4503.0	2997–7826	2.6
V_10Gy_	635.8	396–906	2.3	110.3	58–178	3.1	3855.0	2736–6329	2.3
V_100%_	48.6	30–78	2.6	47.5	25–81	3.3	115.0	65–221	3.4
V_150%_	23.1	14–40	2.9	14.7	6.9–26.9	3.9	–	–	–

The mean of the obtained volumes, the range of their values and the ratio of the highest and lowest individual value are displayed.

### Comparison of volumes between therapies

The prescribed doses were different for the three modalities, consequently the absolute and relative doses also vary. Patients with bigger prostate (more than 60 cm^3^) are not eligible for brachytherapy, furthermore in EBRT extra margin was added around the prostate to get PTV, therefore all average ISVs of the EBRT patients were considerably larger than those of the BT patients. For example, the mean V_100%_ of LDR patients was 2.4 times smaller than that of the EBRT patients, and the ratio was 3.6 for V_1Gy_ (Table 1). Although the V_100%_ values of HDR and LDR therapy did not differ significantly, the average V_10%_ in HDR is 2.0 times larger than that in LDR therapy (Table 1).

### Comparison of chromosome aberrations

**Figure 2:**
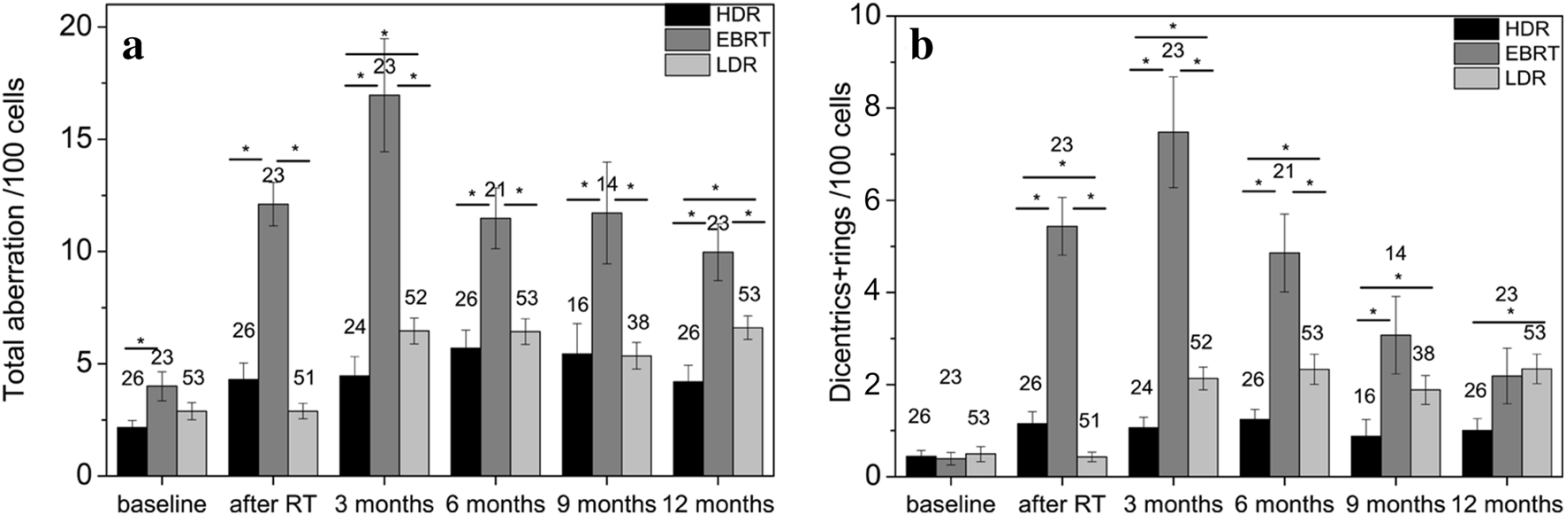
Chromosome aberrations induced by three radiotherapeutic modalities. High dose rate brachytherapy (HDR), external beam radiotherapy (EBRT) and low dose rate brachytherapy (LDR) are studied. (**a**) Total aberrations are shown depending on treatment and follow up time: before therapy (0), immediately after therapy (a.RT), and 3, 6, 9 and 12 months after therapy. (**b**) Dicentrics + rings are displayed. Significant differences (Mann–Whitney test, p < 0.05) are marked with asterisks. Both dicentric plus rings and total aberration values rise after radiotherapy, however, the growth after LDR therapy can be seen only after 3 months. At every timepoint, EBRT group had the highest aberration frequency.

Baseline values of total aberrations obtained prior to the treatment (2.2; 4.0; 2.9 for HDR, EBRT and LDR therapy, respectively) were less than the cutoff limit used in our laboratory for healthy people (5 aberrations/100 cells) in every modality (Fig. 2a). Total aberrations were increased to 4.3; 12.1 and 2.9 total aberrations/100 cells for HDR, EBRT and LDR therapy, respectively (significantly different between HDR and EBRT baseline and post radiotherapy) right after radiotherapy. However, after seed therapy the highest growth was seen in the first 3 months interval (from 2.9 to 6.5 total aberrations/100 cells, p < 0.0001). This is due to the long dose delivery of the low dose rate therapy. The chromosome aberration values are the highest after EBRT therapy in every time point. After 3 months stagnation was observed, except in the EBRT group, where a slow non-significant decrease could be seen (Fig. 2a). Only the total aberration value of the HDR group decreased to baseline levels (5 aberrations/100 cells) during the 1 year follow-up. The dicentrics and ring data showed a similar pattern with lower values (1.2; 5.4 and 0.4 dicentrics + rings/100 cells directly after the therapy for HDR, EBRT and LDR therapy, respectively and 2.1 dicentrics + rings/100 cells for LDR therapy at the third month after the therapy) (Fig. 2b).

### Correlation and regression analysis

We performed correlation analysis in each treatment modality group comparing the ISVs with the biodosimetric values measured at different time points. After Benjamini–Hochberg correction of multiple testing we found that 9 months after LDR therapy the chromosome aberration values (dicentrics + rings, chromatid deletions, total aberration and aberrant cell values) correlated positively and significantly with the largest ISVs, namely V_1%_, V_1Gy_. The correlations were weak, between 0.37 and 0.45 (Table 2). There was no significant correlation at other time points. In HDR therapy, 3 months after radiotherapy dicentric plus rings correlated positively and weakly with most ISVs (Table 3). Aberrant cell number correlated with V_150%_ at this time point as well (Spearman correlation coefficient 0.41). We found no correlations in any other time points of the follow-up. In the EBRT treatment group, the ISVs showed significant positive correlations with various chromosome aberrations directly after the radiotherapy, at the 3rd, 6th and at the 12th month. The aberrations which correlated with the most ISVs are shown in Table 4. Furthermore, the Spearman correlation coefficient for V_10Gy_ are 0.52 and 0.51 for dicentrics + rings and aberrant cells at 3 months, respectively. The frequency of chromatid breaks and aberrant cells correlated with V_10Gy_ (Spearman correlation coefficient 0.50 and 0.56) and aberrant cell number with V_10%_ (0.49) at 6 months. Total aberrations (0.48) and aberrant cell number (0.43) showed correlation with V_10Gy_ at 12 months.

**Table 2:** Spearman correlation coefficients between volumes enclosed by an isodose surface and chromosome aberrations 9 months after LDR brachytherapy.

LDR	At the 9th month after RT (N = 30)			
	Dicentrics + rings	Chromatid deletion	Total aberration	Aberrant cells
V_1%_	0.37		0.41	0.41
V_1Gy_	0.40	0.37	0.45	0.43

*LDR* low dose rate brachytherapy. Dicentrics and rings are the directly radiogen chromosome aberrations, chromosomes with two centomeres or ring shape. Total aberration value is the sum of all aberrations: dicentrics, rings, chromatid or chromosome breaks, translocations and exchanges. All aberration types are customary given per 100 cells.

**Table 3:** Spearman correlation coefficients between volumes enclosed by an isodose surface and dicentrics and rings 3 months after HDR brachytherapy.

HDR	Dicentrics + rings at the 3rd month after RT (N = 24)
V_1Gy_	0.44
V_10%_	0.49
V_10Gy_	0.41
V_100%_	0.48
V_150%_	0.41

*HDR* high dose rate brachytherapy. Dicentrics and rings are the directly radiogen chromosome aberrations, chromosomes with two centomeres or ring shape. All aberration types are customary given per 100 cells.

**Table 4:** Spearman correlation coefficients between volumes enclosed by an isodose surface and chromosome aberrations after EBRT radiotherapy.

EBRT	Directly after RT (N = 23)				At the 3rd month after RT (N = 23)	At the 6th month after RT (N = 21)
	Dicentrics + rings	Chromatid deletion	Total aberration	Aberrant cells	Total aberration	Total aberration
V_1%_	0.59	0.60	0.67	0.70	0.44	0.45
V_1Gy_	0.57	0.53	0.64	0.66		
V_10%_	0.54	0.54	0.62	0.69	0.48	0.47
V_10Gy_	0.54	0.56	0.67	0.77	0.58	0.53

*EBRT* external beam radiotherapy. Dicentrics and rings are the directly radiogen chromosome aberrations, chromosomes with two centomeres or ring shape. Total aberration value is the sum of all aberrations: dicentrics, rings, chromatid or chromosome breaks, translocations and exchanges. All aberration types are customary given per 100 cells.

Univariate regression analysis was performed to explain the variance of chromosome aberrations by variables of ISVs. Multiple regression cannot be used, because of the high collinearity of the variables. We summarized our results after Benjamini–Hochberg correction in Tables 4 and 5. In case of LDR therapy, we found, that none of the ISVs were significant predictors of chromosome aberrations (p > 0.2 R^2^ < 6.4% at the ninth month after therapy). However, at the third month after HDR the predictive value (R^2^) varied between 26.0 and 31.7, the best predictor being V_100%_ (p = 0.004, R^2^ = 31.7%) (Table 5). We also found significant regression models 12 months after the HDR irradiation, where we received no significant correlations. The R^2^ ranged between 15.5% and 20.5% and the concerned aberrations were chromatid breaks, total aberrations and aberrant cell values (Table 5). After EBRT, the data of the regression models of time-points directly after radiotherapy and 9 months later are shown in Table 6. Furthermore, we also found significant regression models at 6 months after the therapy (V_10%_ and chromatid deletions: R^2^ = 19.5) and 12 months after the therapy (dicentrics and rings with V_1%_ and V_1Gy_, R^2^ = 20.1 and 18.7, respectively). On the other hand, less regression relation was found than with rank correlation. R^2^ was between 17.3 and 49.4%, the highest value was calculated for the relationship of V_10%_ and the total aberrations 9 months after the therapy.

**Table 5:** Significant regression values of univariate analysis in case of HDR brachytherapy.

HDR	After 3 months (N = 24)				After 12 months (N = 26)			
Volume	Type	B constant	p value	R^2^	Type	B constant	p value	R^2^
V_1Gy_	Dics + rings	1.54E−03	0.011	26.0	Chromatid d	2.98E−03	0.020	20.5
V_1Gy_					Total a	4.35E−03	0.038	16.7
V_1Gy_					Ab. cells	3.83E−03	0.037	17.0
V_10%_	Dics + rings	2.19E−03	0.006	29.6	Chromatid d	3.68E−03	0.033	17.6
V_10Gy_	Dics + rings	1.89E−02	0.008	28.1	Chromatid d	3.09E−02	0.044	15.8
V_10Gy_					Total a	5.00E−02	0.044	15.8
V_10Gy_					Ab. cells	4.33E−02	0.046	15.6
V_100%_	Dics + rings	4.30E−02	0.004	31.7	Chromatid d	6.53E−02	0.047	15.5
V_150%_	Dics + rings	1.20E+03	0.006	29.3	Chromatid d	2.07E−01	0.028	18.6

Dicentrics and rings are the directly radiogen chromosome aberrations, chromosomes with two centomeres or ring shape. Chromatid deletion is a break in one chromatid strand. Total aberration value is the sum of all aberrations: dicentrics, rings, chromatid or chromosome breaks, translocations and exchanges. Aberrant cell frequency is a number of cells with any aberration. All aberration types are customary given per 100 cells.

**Table 6:** Significant regression values of univariate analysis in case of external beam radiotherapy.

EBRT	Immediately after RT (N = 23)				After 9 months (N = 14)			
Volume	Type	B constant	p value	R^2^	Type	B constant	p value	R^2^
V_1%_	Total a	9.36E−04	0.028	17.3	Chromatid d	1.47E−03	0.009	44.3
V_1%_	Ab. cells	7.33E−04	0.014	25.4				
V_1Gy_	Chromatid d	7.11E−04	0.010	27.8	Chromatid d	1.75E−03	0.012	42.5
V_1Gy_	Total a	1.07E−03	0.040	18.6	Total a	4.15E−03	0.023	36.3
V_1Gy_	Ab. cells	8.48E−04	0.021	22.8	Ab. cells	3.12E−03	0.026	34.9
V_10%_	Chromatid d	1.20E−03	0.007	29.8	Chromatid d	2.28E−03	0.024	35.9
V_10%_	Total a	1.91E−03	0.023	22.3	Total a	6.87E−03	0.005	49.4
V_10%_	Ab. cells	1.52E−03	0.010	27.8	Ab. cells	5.17E−03	0.006	47.8
V_10Gy_	Chromatid d	1.59E−03	0.005	32.4	Chromatid d	2.00E−03	0.042	30.1
V_10Gy_	Total a	2.54E−03	0.017	24.2	Total a	8.42E−03	0.008	45.7
V_10Gy_	Ab. cells	2.06E−03	0.005	31.4	Ab. cells	6.31E−03	0.010	43.7
V_100%_	Chromatid d	2.96E−02	0.011	27.1				
V_100%_	Total a	4.68E−02	0.033	19.9				
V_100%_	Ab. cells	3.63E−02	0.019	23.5				

Dicentrics and rings are the directly radiogen chromosome aberrations, chromosomes with two centomeres or ring shape. Chromatid deletion is a break in one chromatid strand. Total aberration value is the sum of all aberrations: dicentrics, rings, chromatid or chromosome breaks, translocations and exchanges. Aberrant cell frequency is a number of cells with any aberration. All aberration types are customary given per 100 cells.

## Discussion

Brachytherapy, especially LDR therapy is very hard to model in cell cultures because the dose delivery of LDR therapy lasts approximately 1 year. Furthermore, the different distances from the multiple sources and the effect of the blood circulation are hard to reproduce in in vitro conditions. Although the basic knowledge of the laws of radiobiology is also known in this scenario, the summarised impact of the multiple different factors—energy, dose rate, treatment time, irradiated volume—on chromosome aberrations is unknown. In our study, we compared the biological effect of the different radiotherapy modalities of prostate cancer treatment on cellular level, using lymphocytes originate from the blood circulation. With this model, therefore, we could exclude the bias effect of the different irradiated volumes.

Previously, most studies of the research field investigated the effect of organ doses on late radiation toxicities. However, the lymphocytes do not stay in one organ, therefore in biodosimetric investigations organ-independent irradiated volumes should be used. In five studies^19–23^ total reference air kerma (TRAK) was compared with ISVs. The studies followed ICRU 38 recommendation^24^ to use total reference air kerma for the dose and volume description in cervix brachytherapy. On the other hand, these studies applied much higher doses than what we used and they studied these factors in an intracavitary scenario. Furthermore, Barillot et al. showed, the reference volume enclosed by the 60 Gy isodose surface for cervix BT or with combined EBRT to be an independent predictor of the late complications^25^. They demonstrated the relationship between the volumes and rectal complication and soft tissue sequela in univariate and rectal toxicities. As such, the predictive value of ISVs was independent on the mean organ dose^25^.

Lymphocytes die by apoptosis after radiation, which is observed mostly for non-dividing cells like intestinal crypt, in salivary and lacrimal gland cells but rarely for tumor cells. However, most radiation induced cell death occur after an attempted mitosis. Without successful execution, the cells are eliminated by other cell death pathways, such as necrosis or apoptosis, etc.^26^. On the other hand, correlation between spontaneous and radiation induced apoptosis and tumor response was observed^27^ showing the importance of this pathway in therapy outcome as well.

The dose on the lymphocytes can also be important because radiation induces immunological changes. Relevant studies gained importance in the light of the increasingly employed immunotherapies in the patients previously treated with radiotherapy.

Jin et al. developed a model for estimating the “dose on the immune system” in order to investigate its relation to treatment outcome. They proposed that there were rapidly circulating immune cells in the heart, lung and blood vessels and the radiation dose is uniformly delivered to them. On the other hand, there are slowly circulating immune cells in the lymphatic systems and blood reservoirs and they are irradiated only if they are in the irradiated volume at the time of the dose delivery. On 464 non-small cell lung cancer patients they found that higher immune system dose was associated with poorer local progression free survival and overall survival^13^. Ladbury et al. also found the similarly modelled immune dose to be correlated with survival in an independent cohort^15^. To better estimate the “dose on blood” other groups developed computational models^14–17^ as well. These models were able to provide dose distribution information as well. As chromosome aberration technique provides information about the quantity of cells with certain aberration numbers, it reflects dose distribution. Therefore, our data can help to validate similar models in the prostate. Considerations of model modification for brachytherapy can also be tested in our database.

For three treatment modalities of prostate cancer, we calculated volumes irradiated by extremely low doses (minimum was 0.7 Gy) and compared them with chromosome aberrations, which to our knowledge hasn’t been done before. Our work is also a special one with the analysis of LDR and HDR BT as monotherapy by biological dosimetry methods.

Since the HDR therapy (given in a single fraction) and planning were ultrasound based, the field of view was limited. The calculation volume is determined by a user defined distance from the source dwell positions. The maximum distance is 50 mm, and because V_1%_ is beyond this area it cannot be calculated by the treatment planning systems (TPS) (Table 1), therefore was not considered in our study. Also, TPS-s may not calculate the low dose ISVs accurately^28^. Different treatment planning systems would calculate different volumes even on the same image sets, and this may cause higher effect on the larger ISVs. In the case of EBRT, ISVs were retrieved from the TPS. It is also a limitation of our study that various treatment planning systems and multiple techniques were used in dose delivery in EBRT (RapidArc, IMRT, traditional and simultaneous integrated boost), but the small sample size did not allow us to make a subgroup analysis. On the other hand, there is less difference between the isodose volumes in the used different techniques in our cohort, than between the different therapeutic modalities. (The average V_1Gy_ of 3D conformal therapy is 8968 ± 521 cm^3^, it is 7601 ± 511 cm^3^ for IMRT, the t-test is non-significant.) For the same reason, we could not stratify the HDR patients according to the prescribed dose of 19 and 21 Gy. Collection of outcome and toxicity data is still in progress for further analysing clinical differences between the therapies.

The evaluation of volumes in three different kinds of radiotherapy treatments resulted in substantial differences between BT and EBRT regarding both volumes and biological doses (Table 1 and Fig. 2). The explanation of the difference between HDR and LDR therapy in V_10%_ but not in V_100%_ can be the different energies used in HDR or LDR therapies. As the energy of the I-125 (21 keV) is much smaller than that of the Ir-192 (360 keV) the attenuation at a distance of a few centimeters from the source is larger for I-125 that results in a smaller volume irradiated by 10% of the prescribed dose (V_10%_).

We revealed positive correlations between the volumetric features of LDR BT and chromosomal aberrations in blood lymphocytes of patients only at the ninth month after the implantation, but not at other time points (Table 2). However, in the univariate regression analysis, there were no significant predictors of the examined biodosimetric values among the ISVs of different doses. It is important, that although the 98% of the total dose is delivered in LDR BT within 1 year, the dose rate continuously decreases. Furthermore, the description of the radiobiological effect is complicated by the tumour shrinkage and tumour repopulation which can cause complex models^29,30^. Thus, it is not trivial to find linear or quadratic relations. We hypothesize that after 9 months, the irradiation with the decreasing dose rate cannot counteract the death of the damaged lymphocytes, which decreases the aberration frequency. Therefore, the few emerging aberrant cells are cleared out of the blood. However, our results do not exclude any connection between chromosome aberrations and long-term clinical results of LDR BT. In summary, there is a relatively loose link between the chromosome aberrations and ISV-s in the case of LDR BT.

In case of HDR BT, we found that most ISVs correlated positively with radiation specific aberrations (dicentrics + rings) 3 months after the therapeutic intervention (Table 3). All examined volumes turned out to be significant predictors in the univariate regression analysis after 3 months and most ISVs were predictors at 12 months (Table 5). HDR therapy uses higher photon energy compared to LDR therapy, which should cause less DNA damage^31^. The ISVs of absolute doses are also smaller in case of HDR, however, the higher dose rate increases the aberration frequency. These effects cumulatively caused less dicentric + ring frequency in our cohort (Fig. 2). Despite of the lower level of CAs, in HDR therapy, there is still a close relationship between aberration frequency and irradiated volumes. The effect of the volumes was also long-lasting (1 year at least), the durability of the connection was not limited to teletherapy.

In EBRT, most correlations were found immediately after completing the irradiation, but they were observable at the third-, sixth- and at the twelfth-month follow-up time as well (Table 4). Twenty-seven of the variables were observed to be predictors in regression analysis (Table 6). Besides, the highest predictive values (R^2^ = 49.4) were found in EBRT, where the highest irradiated volumes can be found, which may cause a bigger impact on chromosomal aberrations. The strongest significant predictor was found in the case of V_10%_. Although fractionation may eliminate the link between the ISV and biodosimetric values, it is clearly detectable in our results. These results suggest, that some of the lymphocytes may be long-lived and the persistence of chromosome aberrations after radiotherapy was already described^32^.

It may be of interest to note, that in HDR BT mostly dicentrics + rings showed correlations at the third month, which aberrations considered to be radiation specific. However, twelve month after EBRT and HDR BT, not just the radiation specific aberrations were in relation with the volumetric features. We think that these other aberrations also show radiation dose dependence, but not all of them follow linear quadratic dose-dependence, as we published before^33^. Their frequency can also have diverse time dependence as our data measured right after EBRT suggest.

## Conclusion

Relationships between the physical and biological properties of the three therapies were demonstrated, and the strongest were found in case of teletherapeutic treatment. Connections of ISVs and chromosomal aberrations were seen even 1 year after radiotherapy. Our results also suggest, that fractionation do not diminish the connection of ISVs and biological dose. However, sample and data collection for long-time analysis of chromosomal aberrations and toxicity are needed and this work is in progress in our centre.

## Patients and methods

### Patients

One hundred and two patients with low and intermediate prostate adenocarcinoma were recruited from 2015 to 2018 and were followed up for minimum 1 year. BT was offered to patients with prostate volume under 60 cm^3^, with no pubic arch interference and few comorbidities. Their personal preference was also taken into account. After the selection of BT, patients were randomised into the LDR or HDR BT group in the PROMOBRA study, which compares the outcomes of 145 Gy LDR and 1 × 19 or 21 Gy HDR brachytherapy (TC02258087 on Clinicaltrials.gov, for protocol details see below). No patient with previous malignancies or history of radiotherapy was allowed to participate in the study.

### Target volumes and doses

Treatment characteristics are summarised in Table 7. Twenty-three patients were treated with EBRT on a linear accelerator with beam energy of 6–18 MV. The prostate was defined as the first CTV (CTV_pros) for patients with localised, low risk tumour. For intermediate risk patients CTV_pros was extended with 0.5 cm in all direction except for posterior where the rectum is located, plus a 1.0 cm margin was added in cranial direction to define the vesicula seminalis CTV (CTV_PVS). The PTVs were created by the extension of the CTVs with 0.8 cm in all direction. Eight EBRT patients were treated with conventional 2 Gy fractions up to 78 Gy to the prostate (39 fractions), fifteen with simultaneous integrated boost technique (SIB) up to 70 Gy: 2.5 Gy/fraction to the prostate and 2.05 Gy/fraction to the base of the vesicles (28 fractions), both given five times a week (Table 7). EBRT planning was made by Eclipse v13.7 (Varian, Palo Alto, USA) or Pinnacle v9.8 (Philips, Eindhoven, The Netherlands) treatment planning system.

**Table 7:** Numbers of patients according to treatment types and doses.

Therapy	HDR	EBRT	LDR
Low risk	12	3	18
Intermediate risk	14	20	35
Sum	26	23	53

Dose	HDR	EBRT	LDR
19 Gy	17		
21 Gy	9		
70 Gy		15	
78 Gy		8	
145 Gy			53

*BT* brachytherapy, *HDR* high dose rate, *LDR* low dose rate, *EBRT* external beam radiotherapy.

Twenty-six patients received 19 or 21 Gy with HDR BT using afterloading technique with Ir^192^ source and fifty-three received 145 Gy with I^125^ LDR BT (Table 7).

### Implantation technique and treatment planning

The HDR BT treatments were performed in a single fraction in spinal anaesthesia with transrectal ultrasound (US) (Pro Focus 2202; BK Medical ApS, Herlev, Denmark) guidance. A series of axial US images were taken at 5 mm intervals and the treatment planning was performed by Oncentra Prostate 3.2.2. (Elekta Brachytherapy, Veenendaal, The Netherlands) planning system according to the TG43 formalism. Then therapeutic needles were inserted, and both longitudinal and axial planes were used for the needle navigation. The possible prostate movement during insertion was taken into account on the live longitudinal US image, and a new image acquisition was made after the needle insertions. The preplan was copied on the new images, the needle positions and the plan were updated accordingly, both intraoperatively. Finally, an X-ray image was taken for verification purpose and the intraoperative plan was delivered.

For seed implantation stranded seeds with 1 cm separation were used (IsoSeed, I25.S06, Bebig-Theragenics, Berlin, Germany). The implant procedure was similar to that used in HDR brachytherapy. The treatment planning system (TPS) was SPOTPRO 3.1. (Elekta Brachytherapy, Veenendaal, The Netherlands) and TG43 formalism was used. For final dosimetry CT imaging was performed 4 weeks after the implantation and a postimplant plan was made by Oncentra Prostate.

ISVs were determined in Oncentra Prostate for both LDR and HDR therapy. All TPS (including Eclipse and Pinnacle) provide the size of the ISVs after adding the certain isodose lines to the treatment plan. ISVs are not influenced by organ contours, but they are calculated inside the body contour, which are either drawn automatically or by hand. Since chromosome aberration technique sensitively detects the effects of radiation even at 0.1 Gy dose^34^, we presumed that irradiated volumes of small doses such as V_1Gy_ (volume of the body, which was irradiated with the minimum of 1 Gy) could be also important. We also analysed V_150%_, because it is traditionally included in side effect studies.

### Analysis of chromosome aberrations

Along with blood draw for prostate specific antigen determination, heparinised blood for chromosomal aberration measurement was also collected (before radiotherapy, after the treatment and every 3 months for 1 year). In the case of EBRT, blood was taken after the delivery of the last dose, the maximum time frame was 1 h. In the case of BTs, blood was taken on the day after the therapy. Lymphocytes were stimulated with phytohaemagglutinin M (0.2%, Gibco) in RPMI cell culture media (Gibco) and 15% fetal bovine serum on 37 °C. Cell division was arrested with 0.1 μg/ml colcemid (Gibco). Cells were treated with hypotonic solution (75 mM KCl) to swell their volume and fixated five times with 3:1 methanol:acetic acid mixture. The cells were dropped on glass slides in order to produce smears, which were stained with Giemsa solution. Metaphases were scored in regard of chromosomal aberrations: dicentrics and ring chromosomes, chromatid and chromosome breaks, exchanges and translocations were counted. We analysed chromosome aberrations directly and 3, 6, 9, and 12 months after radiotherapy. Our laboratory is harmonized with the ICPEMC^35^ scoring criteria. Chromosome aberrations were counted by two highly experienced cytogenetic assistants, their work was regularly inspected by the study leader.

### Statistics

In the analysis of chromosome aberrations GraphPad Prism 8.0 (GraphPad Software; RRID:SCR_002798) was used for Mann–Whitney tests and IBM SPSS statistics 25.0 for correlation analysis of aberrations and ISVs. As chromosome aberration values are not normally distributed, Spearman correlation analysis was performed considering only significant correlations. Benjamini–Hochberg procedure (0.25 false discovery rate) was applied to handle multiple comparison problem. The results were categorized as follows: moderate correlation was found if 0.50 < correlation coefficient < 0.7 values were calculated and weak correlation was found if the coefficient was ≤ 0.50. As rank correlations are robust against outliers, they were not excluded from the calculations considering that they might be the values of radiosensitive patients and because of the small sample size. Univariate regression (of ISVs and chromosome aberrations) was made in Minitab 18.1 (RRID:SCR_014483) using Assistant tool, which considers both linear and quadratic relationships. We also used the Benjamini–Hochberg procedure (0.25 false discovery rate) on the regression analysis results to discard excess significant connections. Not all patients showed up to every follow up visit, therefore, the number of patients for every analysis was indicated.

### Ethics declarations

All procedures performed in our study involving human participants were in accordance with the ethical standards of the national research committee and with the 1964 Helsinki Declaration and its later amendments. Our patients receiving brachytherapy were participants in the PROMOBRA study (TC02258087 on Clinicaltrials.gov). The EBRT patients were investigated according to the extension of PROMOBRA with the approval of the national Medical Research Council (44179/2013/OTIG and 16738-2/2015/EKU). Informed consent was obtained from all patients.
